# Parental knowledge and attitudes toward genetic counseling and childhood genetic testing for congenital anomalies in Qatar

**DOI:** 10.1002/jgc4.70096

**Published:** 2025-08-20

**Authors:** Houda M. Alkilani, Karen El‐Akouri, Abdulaziz Farooq, Zumin Shi, Mashael Al‐Shafai, Mitchell Stotland, Houssein Khodjet‐El‐khil

**Affiliations:** ^1^ Department of Biomedical Sciences, College of Health Sciences, QU Health Qatar University Doha Qatar; ^2^ Division of Plastic Surgery Sidra Medicine Doha Qatar; ^3^ Division of Genetic and Genomic Medicine Sidra Medicine Doha Qatar; ^4^ Research and Scientific Support Aspetar Doha Qatar

**Keywords:** attitude, congenital anomalies, genetic counseling, genetic testing, parental knowledge

## Abstract

This study aims to evaluate parental knowledge and attitudes toward genetic counseling and testing in the context of pediatric plastic surgery in Qatar. It assesses baseline knowledge to identify educational gaps and factors that may contribute to fear or reluctance in managing children with congenital anomalies. Parents of children with congenital anomalies visiting the pediatric plastic surgery clinic at Sidra Medicine participated in an online questionnaire from October 2022 to February 2023. The 37‐question survey covered demographics, knowledge, and attitudes toward genetic counseling and testing, with knowledge scores ranging from 1 to 12 (scores above 9 indicating high knowledge). Responses were collected from 160 parents, representing various regions: Asia (26.6%), North Africa 25.3%, the Middle East 20.3%, America/Europe 5.7%, and Qatar 22.2%. Among them, 22.9% reported consanguinity, and 37% had children who underwent genetic testing. American/European parents (*p* = 0.016) and those with higher education (*p* = 0.006) showed greater genetic knowledge. Qatari parents had high knowledge 45.7% but lower perceived benefits and higher barriers. Consanguineous parents (*p* = 0.003) and those referred by medical providers (*p* < 0.001) had more positive attitudes toward genetic testing, while those with no prior testing experience or without another child with a genetic disorder displayed negative attitudes. This study highlights the need for culturally appropriate education on genetic counseling and testing for parents of children with congenital anomalies. Genetic counselors should consider education levels and consanguinity when discussing genetic testing to empower parents in making informed decisions.

## 1 INTRODUCTION

Congenital anomalies are a leading cause of morbidity and mortality among infants and children, contributing significantly to lifelong disabilities (Eide et al., 2006; Murphy et al., 2015). These anomalies can arise from various factors, including genetic issues related to chromosomal changes or pathogenic variations in single genes (Brent, 2004). Recent advancements in genomic medicine have improved access to genetic testing, enabling clinicians to molecularly diagnose conditions linked to specific genetic factors. This has elevated the role of genetic counselors, who provide essential medical education and information about genetic risks and testing, alongside clinical and psychosocial support for patients and their families (Ciarleglio et al., 2003; Desrosiers et al., 2019). The implications of genetic testing can be profound, often resulting in a diagnostic label that may be welcomed or not. Therefore, pre‐test genetic counseling is crucial, particularly since children cannot provide informed consent; this responsibility falls to their parents or legal guardians (Lim et al., 2017).

Research shows that parents are more likely to consent to genetic testing for their children when they anticipate clinical benefit (Jamalalail, 2019; Lim et al., 2017; Zhang et al., 2019). Additionally, parental educational levels impact decision‐making in this context (Lim et al., 2017). However, there is limited research examining parental attitudes and knowledge regarding genetic counseling in Middle Eastern populations, particularly in Qatar (Alkuraya & Kilani, 2001; Alsulaiman et al., 2010; Alsulaiman & Hewison, 2007; Jamalalail, 2019; Mohammed et al., 2013). The country has seen demographic changes, with native Qataris making up about 22% of the 2.7 million population (Al‐Dewik et al., 2018). Historically, high rates of consanguinity in the native Qatari community have correlated with increased incidence of autosomal recessive diseases, prompting significant investments in premarital and newborn screening programs (Bener & Alali, 2006; Ministry of Public Health, 2018). Genetic counseling has emerged as a vital component of Qatar's healthcare system, addressing the unique genetic health challenges posed by the nation's demographic characteristics, such as high consanguinity rates and large family sizes. Recognizing its importance, Qatar has developed a wide range of genetic counseling services that cover areas such as premarital screening, reproductive health, prenatal and pediatric care, adult genetics, and oncology. Key healthcare providers, including Hamad Medical Corporation and Sidra Medicine, have played a central role in advancing these services. To support local capacity building, Qatar University introduced a Master's program in Genetic Counseling in 2018. Additionally, the Ministry of Public Health has officially acknowledged genetic counseling as a recognized profession, helping to establish standardized practices and integrate the role within the national health system (Abiib et al., 2024).

Sidra Medicine's Pediatrics Plastic Surgery (PPS) Division is one of the health system's achievements in Qatar that offers high‐quality care for children with congenital and acquired anomalies. In 2018, the division established Qatar's first multidisciplinary clinic for patients with clefts and craniofacial anomalies such as cleft lip +/− palate, craniosynostosis, microtia, etc. The service enables direct internal referrals to clinical genetics and genetic counseling services in cases where patients exhibit multiple phenotypes. Nevertheless, a random observation suggests that only a small proportion of patients at the PPS clinic have undergone genetic counseling or testing; this may be attributed to the workflow established for referring patients to genetic services but also to the knowledge and attitudes of patients parent's regarding the genetic basis of congenital anomalies and genetic testing, which could influence their decisions to consult clinical geneticists and genetic counselors (Abdul Rahim et al., 2020; Al‐Shafai et al., 2022).

This study aimed to assess parents' knowledge and attitudes toward genetic counseling and childhood genetic testing using structured questionnaires. It also explored how perceived benefits and barriers vary across sociodemographic factors such as age, gender, education, and prior exposure to genetic services. Secondary analyses examined these associations in more detail, while exploratory analyses investigated the role of cultural beliefs and the relationship between uncertain responses and overall knowledge levels. The findings are intended to inform the development of targeted interventions that promote the utilization of genetic services, ultimately improving care for children with congenital anomalies.

## 2 STUDY DESIGN AND STUDY PARTICIPANTS

The study involved a cross‐sectional, quantitative interview‐based survey conducted with eligible patients' parents at the Pediatric Plastic Surgery clinic in Sidra Medicine from October 2022 to February 2023. Each parent was given the opportunity to complete the survey individually, with responses collected separately to ensure independent input. However, the data were not treated as paired or analyzed at the couple level; surveys were administered on iPads to consenting parents. The participants were not randomly selected from the general population; they were individuals attending the clinic.

### 2.1 Inclusion criteria

Parents of pediatric patients (ages 1 day to 17 years) attending their first or follow‐up visit at the Pediatric Plastic Surgery clinic in Sidra Medicine between October 2022 and February 2023, specifically those with one or more congenital anomalies, were included.

### 2.2 Questionnaire design and administration

The survey was administered in both English and Arabic, with interpreter support available for non‐speakers; however, all participants were able to complete the survey independently in one of the two provided languages, and no interpreter assistance was ultimately required. The translation from English to Arabic was carried out by a licensed medical translation company, authorized by Sidra Medicine. Respondents filled out the questionnaire using the SurveyMonkey® online tool (https://www.surveymonkey.com/).

The questionnaire (Supplementary Questionnaire S1 and S2) was adapted from previously reported studies in the literature which investigate similar topics using validated questions (Allum et al., 2014; Bíró et al., 2020; Chen et al., 2013; Fitzgerald‐Butt et al., 2010; Henneman et al., 2004, 2013; Levine et al., 2010; Palmer, Martinez, et al., 2009). The questionnaire, reviewed for content relevance and clarity by a board certified genetic counselor, was tailored for participants by the first author, a genetic counseling student, with input from a certified genetic counselor. It included 37 questions divided into six sections. The questionnaire began with demographic questions (1–9), followed by family history inquiries (10–12). It explored prior encounters with genetic counseling and testing (13–16) and assessed parental knowledge about genetics and its role in diseases (17–22) (Table S1). Questions (23–28 and 35) evaluated parental attitudes toward genetic counseling and testing, while questions (29–34 and 36–37) addressed perceived barriers to these services.

For analytical purposes, free‐text responses were organized into categories. Responses to the reason for referral (Table S2) were grouped into three categories: major single/non‐syndromic anomalies (e.g., cleft lip/palate, microtia, and craniosynostosis), major multiple/syndromic anomalies (e.g., Apert syndrome, Crouzon syndrome, and 22q deletion syndrome), and minor anomalies (e.g., ear deformity, syndactyly). For robust statistical analysis, categories were combined into “single anomalies” and “multiple anomalies” due to limited responses for minor anomalies. Questions (14, 16–22, and 25–37) used 5‐point Likert scales (Strongly Agree to Strongly Disagree). For parental knowledge questions (17–22), scores were assigned as follows: 2 for correct answers, 1 for ‘Undecided,’ and 0 for incorrect answers (Chin & Tham, 2020). In our study we applied a method we considered more accurate in assessing respondents knowledge levels, based on methodological rationale and supported by relevant literature (Chin & Tham, 2020; Haga, Barry, et al., 2013). The cumulative knowledge ranged from 0 to 12, categorized as ‘high’ (9–12), ‘moderate’ (6–8), and ‘low’ (below 6). Data from questions (10 and 11) on family history were partially excluded due to low response rates. For perceived benefits (questions 23–28 and 35), scores were assigned as follows: 2 for ‘Strongly Agree’ or ‘Agree,’ 1 for ‘Undecided,’ and 0 for ‘Strongly Disagree’ or ‘Disagree.’ Due to the small sample size, we combined ‘Agree’ with ‘Strongly Agree’ and ‘Disagree’ with ‘Strongly Disagree’ to improve statistical power and clarity. This method is supported by previous studies, including (Olwi et al., 2016).

Parental attitudes were grouped into Agree, Undecided, and Disagree. Higher scores indicated more positive attitudes. For perceived barriers (questions 29–34 and 36–37), scoring was the same, with higher scores reflecting stronger perceived barriers. An open‐ended question was included to allow participants to report additional perceived barriers; however, due to the limited number of responses, these entries were not subjected to thematic analysis.

As the barriers were assessed using Likert‐scale responses rather than binary selections, presenting them as raw frequencies was not feasible; instead, we have described the most commonly endorsed barriers narratively to aid interpretation.

The data analysis utilized SPSS software v28.0. Categorical variables were presented as frequencies and percentages, while continuous variables were tested for normality using the Shapiro–Wilk test and presented as mean ± standard deviation. Parametric tests, such as one‐way analysis of variance (ANOVA) and post hoc pairwise comparisons with Bonferroni correction, were conducted. A chi‐square test determined the association between categorical variables. Pearson's correlation coefficient assessed the correlation between perceived benefits and barriers scores. Multiple linear regression identified factors associated with outcome scores, where only significant factors were included.

A significant level of *p* < 0.05 was used for all statistical analyses.

### 2.3 Ethical approval

Ethical approval was obtained from Qatar University's Institutional Review Board (IRB) under the number: 1967276‐1, as well as from Sidra's IRB under the number: 1916398. Prior to participation, all participants signed a consent form.

## 3 RESULTS

### 3.1 Study participants' demographics

A total of 160 participants filled out the questionnaire. The reason for the referral was mainly due to a major isolated/non‐syndromic congenital anomaly (*n* = 117, 73.1%), followed by major multiple/syndromic anomalies (*n* = 33, 20.6%) and only (*n* = 10, 6.3%) with a minor anomaly (Table S2). Most of the parents who participated in the study were mothers (*n* = 101, 63.1%). The mean age of parents was 37.4 years, and most of the participant ages fell between 30 and 39 years (*n* = 67, 46.2%). In total, 22.2% (*n* = 35) of the participants were Qatari; Asians (*n* = 42, 26.6%) and North Africans (*n* = 40, 25.3%) represented the majority, followed by Middle Eastern (*n* = 32, 20.3%), and Americans/Europeans (*n* = 9, 5.7%). All participant were residents of Qatar with stable employment and housing, reflecting the country's diverse population structure, where expatriates form the majority; only a minority identified as Qatari nationals (Table S3).

Participants with children or other family members with genetic disorders accounted for 12.6% (*n* = 20) of our study sample. About 23% (*n* = 36) of our participants were consanguineous, mostly first cousins 50% (*n* = 18) (Table S3).

### 3.2 Previous experience with genetic counseling and genetic testing

Among the 160 participants, 31.3% (*n* = 50) reported that their child underwent genetic testing in Qatar, while 3.1% (*n* = 5) had testing conducted outside Qatar, and 2.5% (*n* = 4) did not specify the location of the genetic testing. Furthermore, out of the 59 participants whose child had genetic testing in the past, 69% found that genetic testing helped them understand the contribution of genetics to their children's congenital anomaly(ies), while 27.6% were undecided to answer this question, and only 3.4% did not feel any contribution from genetic testing to their understanding of their child's anomaly (Figure S1). In addition, out of 160 participants, 33.8% (*n* = 54) were offered by their medical provider a referral for their child to see a geneticist/genetic counselor. Out of these 54 participants, 72.5% found that the genetic/genetic counseling consultation has helped them understand the contribution of genetics to their child's congenital anomaly(ies), while 23.5% were undecided, and about 4% did not agree (Figure S1).

### 3.3 Parental knowledge of genetics and its contribution to disease/congenital anomalies

The mean parental knowledge score was 8.7 ± 2.0. About 53.7% of participants scored with a high level of knowledge, 40.6% scored with a moderate level (Figures S2 and S3). A significant difference between ethnicities was observed (*p* = 0.016) (Table 1). Post hoc analysis revealed that American and European parents exhibited a higher level of knowledge compared to parents from other groups. Parents with a high school education or lower demonstrated a lower proportion of high knowledge levels compared to those with a Diploma, Graduate level/Master's/PhD, and College/Undergraduate level/Bachelor's education.

**TABLE 1:** Parental knowledge level in association with demographic factors. *p* Significant value <0.05.

Demographic factors	Valid *N*	Low (<6)	Moderate (6–8)	High (9–12)	*p*‐Value
Age of the child (years)					
<2	49	2 (4.1)	23 (46.9)	24 (49.0)	0.347
2 to <7	56	2 (3.6)	19 (33.9)	35 (62.5)	
7 to <12	36	4 (11.1)	17 (47.2)	15 (41.7)	
12 to <18	19	1 (5.3)	6 (31.6)	12 (63.2)	
Sex of the child					
Male	90	4 (4.4)	38 (42.2)	48 (53.3)	0.724
Female	70	5 (7.1)	27 (38.6)	38 (54.3)	
Age of the parent (years)					
<30	19	0 (0.0)	9 (47.4)	10 (52.6)	0.91
30 to <40	67	3 (4.5)	25 (37.3)	39 (58.2)	
40 to < 50	53	3 (5.7)	22 (41.5)	28 (52.8)	
>50	6	0 (0.0)	3 (50.0)	3 (50.0)	
Sex of the parent					
Male	59	4 (6.8)	27 (45.8)	28 (47.5)	0.468
Female	101	5 (5.0)	38 (37.6)	58 (57.4)	
Ethnicity of the parent					
Qatari	35	1 (2.9)	18 (51.4)	16 (45.7)	**0.016**
North African	40	0 (0.0)	15 (37.5)	25 (62.5)	
Middle Eastern	32	4 (12.5)	11 (34.4)	17 (53.1)	
Asian	42	4 (9.5)	21 (50.0)	17 (40.5)	
American and European	9	0 (0.0)	0 (0.0)	9 (100.0)^a^	
Education level of the parent					
High school or less	30	5 (16.7)	17 (56.7)	8 (26.7)^b^	**0.006**
Diploma	16	0 (0.0)	7 (43.8)	9 (56.3)	
College/Undergraduate level/Bachelor's	80	2 (2.5)	26 (32.5)	52 (65.0)	
Graduate level/Master's/PhD	34	2 (5.9)	15 (44.1)	17 (50.0)	
Household income (both parents)					
<5000 QAR	19	2 (10.5)	7 (36.8)	10 (52.6)	0.221
5000–10,000 QAR	42	1 (2.4)	23 (54.8)	18 (42.9)	
10,000–20,000 QAR	46	4 (8.7)	15 (32.6)	27 (58.7)	
>20,000 QAR	50	1 (2.0)	19 (38.0)	30 (60.0)	
How far do you live from Sidra Medicine?					
<30 min	79	3 (3.8)	32 (40.5)	44 (55.7)	0.638
30–60 min	71	6 (8.5)	29 (40.8)	36 (50.7)	
>60 min	9	0 (0.0)	3 (33.3)	6 (66.7)	
Other child or family member with similar congenital anomaly(ies)					
No	136 (87.7)	7 (5.1)	56 (41.2)	73 (53.7)	0.634
Yes	19 (12.3)	2 (10.5)	7 (36.8)	10 (52.6)	
Missing	5				
Other child or family member with a genetic disorder					
No	139 (87.4)	7 (5.0)	61 (43.9)	71 (51.1)	0.112
Yes	20 (12.6)	2 (10.0)	4 (20.0)	14 (70.0)	
Missing	1				
Parental consanguinity					
No	121 (77.1)	8 (6.6)	47 (38.8)	66 (54.5)	0.624
Yes	36 (22.9)	1 (2.8)	16 (44.4)	19 (52.8)	
Missing	3				
Parental consanguinity relationship (*n* = 36)					
1st cousins	18 (50.0)	1 (5.6)	8 (44.4)	9 (50.0)	0.341
2nd cousins	6 (16.7)	0 (0.0)	0 (0.0)	6 (100.0)	
Double 1st cousins	3 (8.3)	0 (0.0)	3 (100.0)	0 (0.0)	
Same Tribe	4 (11.1)	0 (0.0)	2 (50.0)	2 (50.0)	
Not mentioned	5 (13.9)	0 (0.0)	3 (60.0)	2 (40.0)	
Did your child ever undergo a genetic test in the past?					
No	101 (63.1)	6 (5.9)	45 (44.6)	50 (49.5)	0.366
Yes	59 (36.9)	3 (5.1)	20 (33.9)	36 (61.0)	
Did any health care provider ever offer you/your child a referral to genetics/genetic counseling?					
No	106 (66.3)	7 (6.6)	44 (41.5)	55 (51.9)	0.671
Yes	54 (33.8)	2 (3.7)	21 (38.9)	31 (57.4)	
Reason for referral					
Single anomaly	96 (60.0)	1 (1.0)	38 (39.6)	57 (59.4)	0.005
Multiple anomalies	64 (40%)	8 (12.5)	27 (42.2)	29 (45.3)	

^a^ After post hoc comparison, the parents who were American or European were more likely to have a knowledge score >9 compared to others (*p* < 0.05).

^b^ After post hoc comparison, the parents with a high school education level were less likely to obtain a high knowledge score (9+) compared to parents with diploma, undergraduate, and postgraduate education levels (*p* < 0.05).

Although we did not collect data on the source or content of participants' education, our aim was to evaluate whether the level of education influenced openness to scientific information and proactive health‐seeking behavior, particularly in the context of increasing accessibility to health‐related knowledge.

### 3.4 Parent's perceived benefits toward genetic counseling and genetic testing

The data obtained shows that 74.7% of the participants said that they would be open to consider genetic testing for their child if it were suggested by a healthcare provider, whereas 13.9% said they would not, and 11.4% were undecided. Similarly, the majority 70% of parents who replied to the study said they would be open to being referred to genetic counseling if they had not already been, 13.8% said they would not be open to a referral, and 16.3% were not sure (Figure S4). Furthermore, most respondents above 85% were in favor of agreeing with perceived benefits, with the exception of the question asking if genetic test results are always accurate, where 41% agreed, 50% were undecided, and only 8.9% disagreed (Figure S5).

### 3.5 Association between parent's perceived benefits and demographic factors

We examined correlations between demographic factors and participants' attitudes toward the benefits of genetic counseling and testing. The data showed that parental consanguinity (*p* = 0.003), prior genetic testing for children (*p* = 0.009), and referrals to genetic counseling by medical providers (*p* = 0.001) significantly influenced positive attitudes (Table 2).

**TABLE 2:** Attitudes toward genetic counseling and genetic testing (perceived benefits) and (perceived barriers) versus demographic factors. *p* Significant value <0.05.

Attitude	Perceived benefits			Perceived barriers		
Demographic factors	*N*	Mean score ± SD	*p*‐Value	*n*	Mean score ± SD	*p*‐Value
Age of the child (years)						
<2	49	12.3 ± 2.0	0.102	49	5.6 ± 3.3	0.112
2 to <7	56	12.2 ± 1.7		56	5.2 ± 2.9	
7 to <12	36	11.6 ± 2.2		36	4.8 ± 3.2	
12 to <18	19	11.2 ± 2.4		19	7.0 ± 4.7	
Sex of the child						
Male	90	12.2 ± 1.8	0.188	90	5.8 ± 3.4	0.086
Female	70	11.7 ± 2.3		70	4.9 ± 3.3	
Age of the parent (years)						
<30	19	13.0 ± 1.3	0.074	19	5.9 ± 3.1	0.659
30 to <40	67	11.8 ± 2.1		67	5.1 ± 3.3	
40 to <50	53	12.1 ± 1.9		53	5.1 ± 3.1	
>50	6	11.0 ± 1.9		6	6.2 ± 5.6	
Sex of the parent						
Male	59	12.2 ± 1.8	0.252	59	5.9 ± 3.5	0.129
Female	101	11.8 ± 2.1		101	5.1 ± 3.3	
Ethnicity of the parent						
Qatari	35	11.8 ± 2.1	0.482	35	6.5 ± 4.2	**0.028** *
North African	40	12.2 ± 1.9		40	5.9 ± 2.9	
Middle Eastern	32	12.4 ± 2.0		32	5.0 ± 3.0	
Asian	42	11.6 ± 2.2		42	5.0 ± 3.3	
American and European	9	12.0 ± 1.9		9	2.8 ± 1.9	
Education level of the parent						
High school or less	30	12.2 ± 2.1	0.658	30	6.4 ± 4.0	0.237
Diploma	16	12.3 ± 2.4		16	4.6 ± 2.1	
College/Undergraduate level/Bachelor's	80	12.0 ± 2.1		80	5.1 ± 3.3	
Graduate level/Master's/PhD	34	11.6 ± 1.5		34	5.6 ± 3.4	
Household income (both parents)						
<5000 QAR	19	12.3 ± 2.1	0.481	19	4.2 ± 2.8	0.372
5000–10,000 QAR	42	12.3 ± 1.8		42	5.3 ± 3.5	
10,000–20,000 QAR	46	12.0 ± 2.1		46	5.8 ± 3.0	
>20,000 QAR	50	11.7 ± 1.9		50	5.4 ± 3.8	
How far do you live from Sidra Medicine?						
<30 min	79	12.1 ± 2.0	0.599	79	5.3 ± 3.7	0.844
30–60 min	71	11.8 ± 2.1		71	5.6 ± 3.1	
>60 min	9	12.4 ± 1.6		9	5.1 ± 2.7	
Other children or family member with similar congenital anomaly(ies)						
No	136 (87.7)	11.9 ± 2.1	0.560	136 (87.7)	5.4 ± 3.3	0.637
Yes	19 (12.3)	12.2 ± 1.4		19 (12.3)	5.1 ± 3.5	
Missing	5			5		
Other child or family member with a genetic disorder						
No	139 (87.4)	11.9 ± 2.1	0.443	139 (87.4)	5.6 ± 3.5	0.018
Yes	20 (12.6)	12.3 ± 1.5		20 (12.6)	3.8 ± 2.1	
Missing	1			1		
Parental consanguinity						
No	121 (77.1)	11.8 ± 2.0	**0.003**	121 (77.1)	5.4 ± 3.5	0.804
Yes	36 (22.9)	12.8 ± 1.4		36 (22.9)	5.3 ± 3.1	
Missing	3			3		
Did your child ever undergo a genetic test in the past?						
No	101 (63.1)	11.7 ± 2.1	**0.009**	101 (63.1)	6.1 ± 3.6	**<0.001**
Yes	59 (36.9)	12.5 ± 1.8		59 (36.9)	4.2 ± 2.4	
Did any health care provider ever offer you/your child a referral to genetics/genetic counseling?						
No	106 (66.3)	11.5 ± 2.2	**<0.001**	106 (66.3)	5.8 ± 3.6	**<0.001**
Yes	54 (33.8)	12.8 ± 1.3		54 (33.8)	4.6 ± 2.8	
Reason for referral						
Single anomaly	96 (60.0)	9.0 ± 1.8	0.132	96 (60.0)	5.5 ± 3.4	0.856
Multiple anomaly	64 (40%)	8.4 ± 2.1		64 (40.0)	5.4 ± 3.4	

* After post hoc comparison, parents who were of American or European ethnicity scored significantly lower on perceived barriers score compared to Qatar nationals (*p* = 0.02).

### 3.6 Association between parent's perceived benefits and knowledge level

There were no significant differences between knowledge levels and attitude scores (*p* = 0.349) (Figure S6). A correlation analysis showed no significant correlation between attitude scores and knowledge scores (*r* = 0.067, *p* = 0.400) (Figure S8).

### 3.7 Parent's perceived barriers toward genetic counseling and genetic testing

A substantial majority of respondents above 55% expressed disagreement with most proposed barriers, except for inquiries regarding the cost of genetic testing and the potential impact on private insurance. Approximately 50% of respondents were undecided, and about 47% think that genetic testing is expensive (Figure S9).

### 3.8 Association between parent's perceived barriers and demographic characteristics

The data revealed that the ethnicity of the parent (*p* = 0.028) was associated with perceived barriers; post hoc testing confirmed Qatari parents exhibited the highest score with negative attitudes compared to other ethnicities (*p* = 0.031) (Table 2).

### 3.9 Correlation between parent's perceived barriers and knowledge level

No significant association between knowledge level and barrier perception scores (*p* = 0.058) was identified (Figure S10). Upon conducting a correlation analysis between knowledge scores and barrier scores, the plot illustrated a negative correlation (*r* = −0.157, *p* = 0.048) suggesting higher knowledge score when compared to lower barrier scores (Figure S12).

## 4 DISCUSSION

This study explores the knowledge and attitudes of parents of children with congenital anomalies attending the Pediatric Plastic Surgery Clinic at Sidra Medicine in Qatar, specifically in relation to genetic counseling and testing. The cohort studied is more representative of the broader population accessing tertiary care services at Sidra Medicine, rather than the Qatari national population specifically. Therefore, the findings should be interpreted within this context. While the study provides valuable insights into the healthcare setting in Qatar, its generalizability to all Qatari residents is limited due to the country's highly diverse and multiethnic population.

Half of the participants had an undergraduate degree, with 36.9% reporting their child had undergone genetic testing and 33.8% referred for counseling. Among those tested, 69.0% found genetic testing beneficial, while 72.5% found counseling helpful, indicating broad acceptance of these resources, similar to findings from Ontario and the United States (Etchegary et al., 2010; Saylor et al., 2019).

While a statistically significant difference was observed in the distribution of referral reasons (*p* = 0.005), this was not deemed to have substantial clinical relevance in the context of our study objectives, which centered on parental knowledge and attitudes toward genetic counseling and testing.

### 4.1 Knowledge level and impact of demographic factors

A recent study on Qataris' attitudes toward genetic testing and participation in the Qatar Genome Project reported a participation knowledge rate of 56.1% (Abdul Rahim et al., 2020), comparable to the 53.7% found in this study. Research in Malaysia and China indicated that education level, field of study, higher income, and prior exposure to genetic testing significantly influence genetic knowledge (Chin & Tham, 2020; Zhang et al., 2020). Contrary to expectations, our results showed no enhancement in genetic knowledge from prior counseling or testing, possibly due to our focus on basic genetic principles rather than specific details.

Unlike a Saudi study that found links between genetic knowledge and demographic factors (Alotaibi et al., 2022), we identified no such associations; possibly due to our smaller sample size.

Our findings showed that parents of American and European descent demonstrated higher levels of genetic knowledge compared to those from Asian, Qatari, Middle Eastern, and African backgrounds. This highlights the need for culturally sensitive genetic counseling, particularly in diverse populations with differing beliefs and levels of familiarity with genetics. In this study, cultural sensitivity refers to an understanding of sociocultural factors relevant to the Qatari context, including language preferences, religious values, health‐related beliefs, and norms around family‐centered decision‐making. These considerations were indirectly incorporated into the questionnaire design to ensure cultural appropriateness. It is also important to recognize that native Qatari and Middle East and North Africa (MENA) populations may hold different cultural views from Asian and Western expatriates—differences that can influence attitudes toward genetic services, as noted in regional literature. Furthermore, a systematic review and related study emphasize that cultural beliefs significantly shape the comprehension and acceptance of genetic information in low‐ and middle‐income countries (Khoury et al., 2007; Zhong et al., 2021). This underscores the importance of addressing cultural factors to enhance the accessibility and effectiveness of genetic services in the region.

### 4.2 Parents' attitudes toward perceived benefits of genetic counseling and genetic testing

Parents generally exhibit positive attitudes toward genetic counseling and testing, with 70% willing to be referred for counseling, even if they had not previously sought it. This aligns with findings from a Qatari study on attitudes toward genetic testing and the Qatar Genome Project (Abdul Rahim et al., 2020). Within the cultural context of Qatar and the broader Gulf region, patients often show a strong inclination to defer to medical authority, which may contribute to the high acceptance of genetic testing when recommended by healthcare professionals. In our study, 74% of parents reported a willingness to proceed with testing for their child if advised by a healthcare provider. This contrasts with findings from a United States‐based study, where only 46% of parents of deaf children pursued genetic testing despite recommendations from pediatricians (Palmer, Lueddeke, & Zhou, 2009); these differences underscore the pivotal role of trusted healthcare professionals in shaping parental decision‐making in this region.

Our analysis identified two demographic factors significantly linked to positive attitudes toward genetic counseling: parental consanguinity (*p* = 0.003) and prior healthcare referrals (*p* < 0.001). To support our finding from the same region, a Saudi study revealed that individuals from consanguineous marriages tended to have favorable attitudes toward genetic testing (Alotaibi et al., 2022), with 80.2% aware of the increased risk of genetic disorders in their community (Alnaqeb et al., 2018). This increased awareness within Qatar may be attributed to initiatives like the Qatari National Newborn Screening program established in 2003 and the National Premarital Screening program initiated in 2009 (Al‐Dewik et al., 2018).

Prior referrals from medical providers were also associated with a positive attitude toward genetic counseling; to support this finding, a US study indicated that parents referred by physicians demonstrated greater knowledge of genetics and a more favorable view of genetic testing for their children (Haga, Burke, & Agans, 2013).

Our findings did not show a significant correlation between parental knowledge levels and attitude scores regarding the perceived benefits of genetic counseling. These results contrast with a Chinese study that found a partial significance between knowledge and attitudes toward genetic testing (Zhang et al., 2020). Another Qatari study showed students had higher knowledge and positive attitudes toward premarital screening (Al‐Shafai et al., 2022). Additionally, a study in North Carolina revealed a persistent lack of understanding of genetic concepts, even among educated individuals (Haga, Barry, et al., 2013), underscoring the need for enhanced education on the benefits and limitations of genetic testing.

Cultural and social perceptions significantly influence attitudes toward genetic testing. A study in Jordan involving 1149 participants indicated a generally positive view of genetic testing but revealed gaps in understanding gene‐related concepts versus disease‐related knowledge (Khdair et al., 2021). This highlights the necessity for improved public awareness to enable informed decision‐making in pursuing genetic testing.

In our study, the lack of correlation between parents' knowledge and perceived benefits of genetic counseling may stem from a small sample size. When we looked at the knowledge questions, we noted that correctly defining congenital anomalies was associated with a more positive attitude toward genetic counseling (*p* = 0.001). This indicates that improving parents' understanding of congenital anomalies definition is vital for promoting favorable attitudes toward genetic counseling and testing. This suggests that improving parents' understanding of congenital anomalies is vital for fostering favorable attitudes toward genetic counseling and testing. Additionally, it is crucial for physicians to identify patients who may benefit from genetic testing and counseling and provide appropriate referrals, thereby enhancing families' willingness to pursue these services.

### 4.3 Parents' attitudes toward perceived barriers to genetic counseling and genetic testing

Perceptions of barriers to genetic counseling and testing could be influenced by various factors, including cultural beliefs, access to healthcare, and personal experiences with genetic testing. In our study, over 55% of respondents disagreed with proposed barriers to these services. After accounting for confounding variables, we identified three significant factors that shape attitudes toward perceived barriers: ethnicity, family history of genetic disorders, and prior genetic testing experiences.

Ethnicity plays a crucial role in shaping perceptions of barriers to genetic counseling and testing. Different ethnic groups encounter distinct cultural, social, and economic challenges that can affect their willingness to engage with these services. Our research indicated that respondents from the United States and Europe reported fewer barriers compared to those from Qatar, North Africa, and Asia. This aligns with a systematic review which found that a majority of participants from diverse backgrounds in the United States were willing to participate in genetic research (Fisher et al., 2020); conversely, earlier studies have shown that African American women often harbor negative attitudes toward genetic testing for breast cancer, stemming from distrust in healthcare systems and fears of discrimination, along with cultural beliefs about illness (Halbert et al., 2012). Similarly, a systematic review focusing on Asian American attitudes noted that language barriers contributed to lower communication rates about genetic risks within families (Young et al., 2021). These findings underscore the necessity for genetic counselors to offer culturally sensitive approaches that are tailored to various ethnic groups.

The family history of genetic disorders significantly impacts perceptions regarding genetic counseling and testing. Individuals with a family member affected by a genetic disorder tend to have a more positive outlook toward these services, likely due to their heightened awareness of potential benefits and risks. For instance, a study in Saudi Arabia revealed that individuals with such family histories viewed genetic testing as a valuable tool for disease prevention (Alotaibi et al., 2022).

Personal experiences with genetic testing also correlate with attitudes toward genetic counseling. Those who have previously undergone testing generally perceive fewer barriers, suggesting that education and familiarity can influence perceptions positively. This highlights the importance of providing accessible educational resources related to genetic counseling and testing.

When assessing various factors as barriers, our findings revealed that 24% of participants viewed health insurance as a hurdle, with 52.9% remaining undecided. This uncertainty may be attributed to the relatively recent introduction of health insurance in Qatar, where many individuals lack private insurance or understanding of how it functions. Additionally, 46.8% of parents identified costs as a significant barrier, consistent with earlier studies (Lemke et al., 2022; Rahma et al., 2020).

Religious beliefs are known to influence attitudes toward genetic services (Likhanov et al., 2023). In Qatar's context, where the majority of the population, 95%, identifies as Muslim, concerns about the religious implications of genetic testing may arise. Although we did not directly ask participants to state their religion, the survey included a question on whether their religious beliefs affected their attitudes toward genetic counseling and testing. Notably, only 5.8% of respondents indicated that religious beliefs would deter them from seeking such services. This aligns with prior regional literature, which highlights that while religion may not be a primary barrier, it can shape perspectives in more nuanced ways. A global systematic review with other regional studies has identified concerns such as stigma, fatalism (Qadar), consanguinity, and ethical discomfort with reproductive technologies as common religiously influenced factors in Muslim‐majority settings (Majeed‐Saidan et al., 2014; Tadmouri et al., 2009; Zhong et al., 2021). These findings were considered when interpreting responses within the broader cultural and religious context of Qatar. Concerns about genetic testing's impact on family relationships were noted, with 15.4% of parents agreeing that it could cause family conflict. Privacy concerns were also evident, with 16.8% of parents fearing that their privacy would not be respected. Furthermore, 16.5% expressed worries about potential social stigma associated with genetic disorders.

Interestingly, our study did not find a significant association between knowledge level and attitudes toward perceived barriers (*p* = 0.058). However, a negative correlation was observed, indicating that higher knowledge scores are related to lower perceived barriers (*p* = 0.048). This suggests that misinformation about genetic risks and misconceptions about genetic disorders contribute to perceived barriers.

Ultimately, our findings highlight the need for targeted interventions to address perceived obstacles to genetic counseling and testing in Qatar. By focusing on issues such as cost, privacy, and education, healthcare providers and policymakers can enhance access to genetic services and improve health outcomes for families. Addressing these barriers is essential for fostering a supportive environment that encourages individuals to engage with genetic counseling and testing.

## 5 CONCLUSION

The study's implications are significant for parents, healthcare providers involved in clinical genetics, genetic counseling, and genetic testing. While the focus is on parents of children with congenital anomalies, the insights can extend to parents of children with any medical condition with potential genetic roots. The diverse geographical backgrounds of participants suggest the need for additional education and support, especially for parents from Qatar, North Africa, the Middle East, and Asia. Addressing barriers, such as lack of time, knowledge gaps, stress, and cultural factors, is essential. Culturally sensitive and tailored education is crucial, ensuring accurate information is provided, addressing perceived benefits and barriers. Collaboration between healthcare providers and genetic counselors is emphasized for optimal care in pediatric plastic surgery, ensuring every patient receives comprehensive support and the best possible outcomes.

### 5.1 Limitations

This study, examining genetic counseling and testing in Qatar, offers valuable insights into the experiences and attitudes of individuals and families dealing with potentially genetically influenced medical conditions. However, we recognize that the relatively small and non‐representative sample could have biased the statistical analysis and limited particularly the correlation between attitudes and demographic or knowledge factors. The participants are parents of children with congenital anomalies that necessitate plastic surgery. This makes our study sample not representative of whole pediatric patients, which could have limited and influenced perception of benefits and barriers of parents to what is related to the type of their children's anomalies.

The list of barriers included in the questionnaire was guided by both regional and international literature; however, not all items were specifically drawn from studies involving Qatari individuals, which may affect the cultural relevance of certain response options. A key limitation of this study is that, although the survey tool was adapted from existing sources, it was not based on a previously validated instrument. Consequently, findings related to participants' knowledge and attitudes should be interpreted with caution. Furthermore, the limited number of survey questions may have restricted the depth of insights and introduced potential response bias. While we aimed for brevity to encourage participation, a more comprehensive questionnaire might have yielded richer data but could have adversely affected response rates due to increased participant burden. Lastly, the exclusive focus on parental perspectives underscores the importance of future research adopting a more comprehensive approach, involving healthcare providers, policymakers, and other stakeholders' integral to the genetic counseling and testing services delivery.

## AUTHOR CONTRIBUTIONS

**Houda M. Alkilani:** Formal analysis; investigation; methodology; project administration; resources. **Karen El‐Akouri:** Mentorship and revision. **Abdulaziz Farooq:** Analysis. **Zumin Shi:** Analysis. **Mashael Al‐Shafai:** Mentorship and revision. **Mitchell Stotland:** Mentorship and revision. **Houssein Khodjet‐El‐khil:** Mentorship; analysis; and revision.

## ETHICS STATEMENT

Human studies and informed consent: This study was reviewed and granted exemption by the Sidra Medicine and Qatar University Institutional Review Board. Informed consent was obtained from all individual participants before initiating the survey.

Animal studies: No non‐human animal studies were conducted by the authors for this article.

## Supporting information


Data S1

## Data Availability

The data that support the findings of this study are available on request from the corresponding author. The data are not publicly available due to privacy or ethical restrictions.
